# Preclinical Therapy of Disseminated HER-2^+^ Ovarian and Breast Carcinomas with a HER-2-Retargeted Oncolytic Herpesvirus

**DOI:** 10.1371/journal.ppat.1003155

**Published:** 2013-01-31

**Authors:** Patrizia Nanni, Valentina Gatta, Laura Menotti, Carla De Giovanni, Marianna Ianzano, Arianna Palladini, Valentina Grosso, Massimiliano Dall'Ora, Stefania Croci, Giordano Nicoletti, Lorena Landuzzi, Manuela Iezzi, Gabriella Campadelli-Fiume, Pier-Luigi Lollini

**Affiliations:** 1 Department of Experimental, Diagnostic and Specialty Medicine, University of Bologna, Bologna, Italy; 2 Department of Pharmacy and Biotechnology, University of Bologna, Bologna, Italy; 3 Rizzoli Orthopedic Institute, Bologna, Italy; 4 CESI Aging Research Center, G. D'Annunzio University, Chieti, Italy; Harvard Medical School, United States of America

## Abstract

Oncolytic viruses aim to specifically kill tumor cells. A major challenge is the effective targeting of disseminated tumors in vivo. We retargeted herpes simplex virus (HSV) tropism to HER-2 oncoprotein p185, overexpressed in ovary and breast cancers. The HER-2-retargeted R-LM249 exclusively infects and kills tumor cells expressing high levels of human HER-2. Here, we assessed the efficacy of systemically i.p. delivered R-LM249 against disseminated tumors in mouse models that recapitulate tumor spread to the peritoneum in women. The human ovarian carcinoma SK-OV-3 cells implanted intraperitoneally (i.p.) in immunodeficient Rag2^−/−^;Il2rg^−/−^ mice gave rise to a progressive peritoneal carcinomatosis which mimics the fatal condition in advanced human patients. I.p. administration of R-LM249 strongly inhibited carcinomatosis, resulting in 60% of mice free from peritoneal diffusion, and 95% reduction in the total weight of neoplastic nodules. Intraperitoneal metastases are a common outcome in breast cancer: i.p. administration of R-LM249 strongly inhibited the growth of ovarian metastases of HER-2+ MDA-MB-453 breast cells. Brain metastases were also reduced. Cumulatively, upon i.p. administration the HER-2-redirected oncolytic HSV effectively reduced the growth of ovarian and breast carcinoma disseminated to the peritoneal cavity.

## Author Summary

In the genome of human herpes simplex virus (HSV) we have replaced part of the sequences encoding the receptor-binding glycoprotein with antibody fragments directed against the oncoprotein HER-2, overexpressed in human breast and ovarian cancers. The retargeted HSV only infects and kills human cancer cells expressing high levels of HER-2. Earlier experiments showed that the retargeted HSV injected inside localized tumors inhibits human tumor growth in immunodeficient mice. As tumor dissemination is the major cause of cancer mortality, we have now used the HER-2-retargeted HSV to treat disseminated HER-2+ ovarian and breast cancer in immunodeficient mice. Intraperitoneal treatments significantly inhibited the peritoneal spread (carcinomatosis and ascites formation) of ovarian cancer cells and the metastatic growth of breast cancer cells in the ovaries. Brain metastases were also inhibited. Our results showed that a HER-2-redirected oncolytic HSV is an effective therapeutic agent against metastatic HER-2^+^ cancers and, more generally, provide the first demonstration of the efficacy of a systemically-administered, retargeted oncolytic HSV.

## Introduction

The past decades have witnessed remarkable progresses in the ability to treat numerous cancers by means of surgery, chemio- and radiotherapy, or combinations thereof. Nonetheless, there remains a tremendous burden of tumors not sensitive or accessible to standard treatments. Oncolytic virotherapy exploits the intrinsic ability of viruses to kill the target cell and simultaneously to spread to other target cells. A key requirement is that the virus specifically targets cancer cells [1]. Herpes simplex virus- 1 (HSV-1) is being actively investigated in preclinical and phase 1–3 clinical studies as it lends itself to numerous genetic modifications that make it cancer-specific [2], [3].

The strategy pursued in our laboratories is to modify HSV-1 tropism, and efficiently retarget the virus to cancer-associated cell-surface molecules, such as the human epithelial growth factor receptor 2 (HER-2), a member of the tyrosine kinase receptors [4]–[9]. The clinical impact of the HER-2 oncogene stems from the fact that it is overexpressed in human breast and ovary carcinomas (>200,000 new cancer cases each year in the U.S.), and correlates with worsened prognosis. Because of these properties, HER-2 is currently the target of antibody-based (trastuzumab) therapies, or small molecule tyrosine kinase inhibitors.

We took advantage of the fact that HSV encodes a multipartite fusion/entry apparatus made of four essential glycoproteins, and that one of these, gD, is the major determinant of the viral tropism [10]. Replacement of the Ig-folded gD core with a single chain antibody to HER-2 subverts the viral tropism, and enables HSV to selectively infect HER-2+ cancer cells and spare the usual targets. By this strategy, the killing capacity of wt-virus is fully preserved, and the high safety profile has not been achieved at the expenses of replication, as was the case in earlier oncolytic-HSV (o-HSV) which were deleted of virulence genes, and overall attenuated in replication capacity [11], [12]. Previous studies from our laboratories showed that the HER-2-retargeted R-LM249 exerts antitumor activity when administered intratumorally to nude mice bearing HER-2-hyperexpressing human tumors [6]. A single injection dramatically impaired tumor growth. Repeated injections of R-LM249 doses higher than 10^6^ pfu left 60% of mice tumor-free, and dramatically reduced the tumor size in the remaining 40%. Recently, a HSV retargeted to HER-2 showed efficient antitumor activity in an intracranial model of high-grade glioma in immunosuppressed and immunocompetent mice [7], [9]. Overall, these findings demonstrated that R-LM249 is effective in killing HER-2+ tumors in vivo, when administered locally [6].

The major clinical problem of HER-2+ neoplasms is disseminated disease, not the primary tumors which are usually removed surgically, coupled to the development of cells resistant to the biological inhibitors. Ovarian cancer disseminates within the peritoneal cavity, thus hampering surgical resection, and frequently produces voluminous peritoneal ascites [13]. Breast cancer disseminates systemically, most frequently to lungs, peritoneal organs, bones, brain in addition to peritoneal cavity [14]. Here we performed the preclinical evaluation of i.p. administered R-LM249 against peritoneal metastases of human ovarian and breast cancers in murine hosts. We show that the i.p.-administered HER-2-redirected HSV effectively inhibits peritoneal dissemination of HER-2+ ovarian and mammary carcinomas.

## Results

### In vitro activity of R-LM249 and trastuzumab against ovary and mammary cancer cells

**Figure 1:**
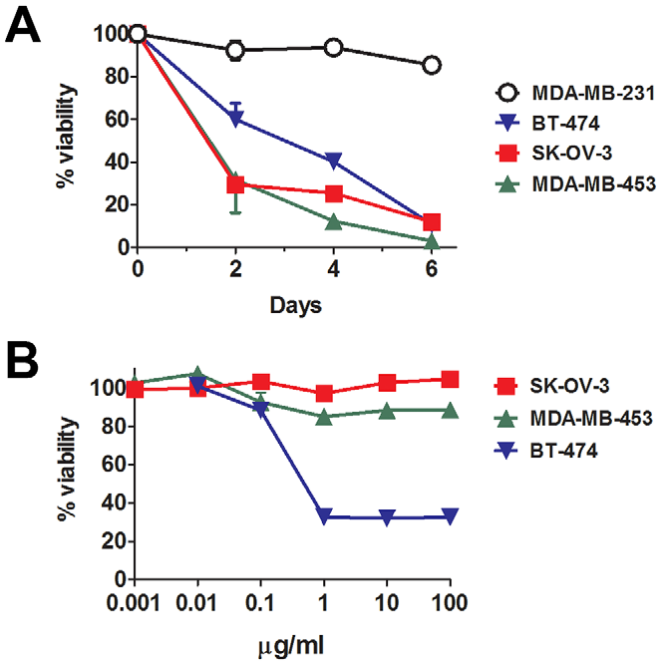
*In vitro* sensitivity to HER-2-redirected HSV and to trastuzumab. (**A**) Cytotoxicity of R-LM249 for cells with high (SK-OV-3, MDA-MB-453 and BT-474) or very low/negative (MDA-MB-231) HER-2 expression. Cells were infected with R-LM249 (10 pfu/cell). Cell viability was measured at the indicated days after infection, by alamarBlue assay. Each point represents the average of quadruplicates + SD expressed as percentage with respect to uninfected cells. (**B**) Effect of trastuzumab after 72 hr culture. Mean and SEM from 3–5 independent experiments is shown.

*In vitro* R-LM249 killed all the HER-2^+^ human cancer cells lines, both of ovary (SK-OV-3) and mammary (MDA-MB-453 and BT-474) origin, but not the HER-2-low/negative MDA-MB-231 cells (Fig. 1A). Microscopical examination of R-LM249-treated BT-474 and SK-OV-3 cultures revealed some residual cells, possibly accounting for 10% viability reported in the graph at 6 days. When such cells were cultured they were unable to proliferate, and therefore most likely already committed to death. Of note, SK-OV-3 and MDA-MB-453 cells are resistant to the growth inhibition by trastuzumab (Fig. 1B) [15]. Thus, HER-2-redirected HSV is effective against HER-2+ trastuzumab-resistant cells, an important property in view of clinical application of R-LM249.

### Therapy of peritoneal carcinomatosis of HER-2+ ovarian carcinoma

**Figure 2:**
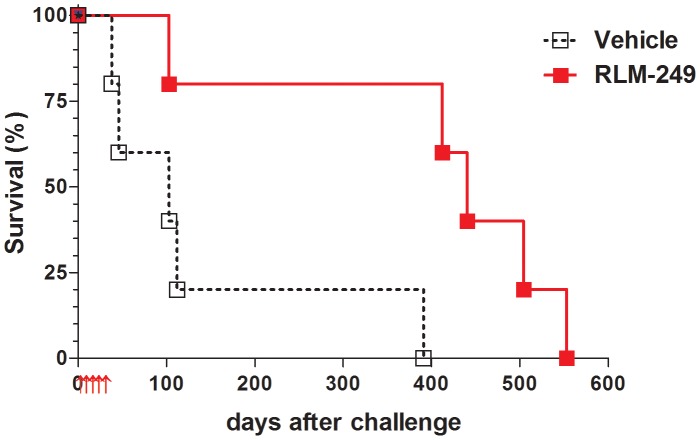
Therapy with the HER-2-redirected oncolytic HSV, R-LM249, of *nude* mice bearing i.p. human SK-OV-3 ovarian carcinoma. Red arrows below *x*-axis mark the days in which groups of 5 mice received weekly i.p. treatments with R-LM249 (2×10^7^ p.f.u./treatment) or vehicle (PBS). Significance of curve comparison: p = 0.02 by the Mantel-Haenszel test.

Previously, we showed that R-LM249 exerts anti-tumor activity against subcutaneously-injected human ovary cancer cells. Here, we investigated the therapeutic activity of R-LM249 against the intraperitoneal (i.p.) growth of SK-OV-3 cells. In a first set of experiments, SK-OV-3 cells were injected i.p. in athymic nude mice. Fig. 2 shows that repeated i.p. administrations of R-LM249 significantly delayed the growth of SK-OV-3 peritoneal carcinomatosis, resulting in a prolongation of median survival time from 103 to 440 days. The majority of treated mice died when they reached the lifetime expected for nude mice, not because of xenograft growth. It can be seen that the growth of SK-OV-3 tumors in nude mice was very slow, in agreement with previous reports [16], and the whole experiment depicted in Figure 2 took one and a half year, clearly hindering further investigations in this model system.

**Figure 3:**
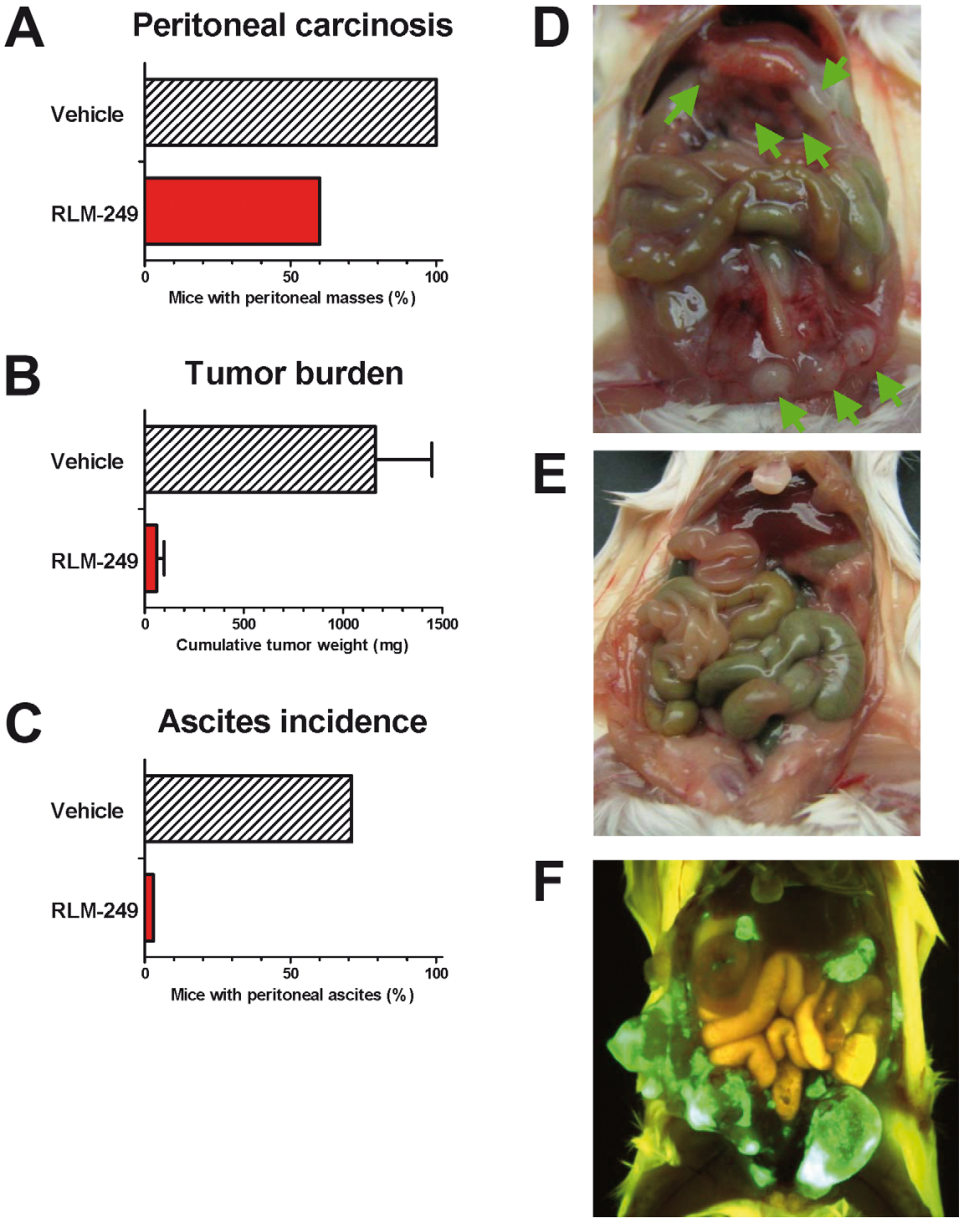
Therapy with R-LM249 of Rag2^−/−^;Il2rg^−/−^ mice bearing i.p. human SK-OV-3 ovarian carcinoma. (**A–C**) incidence of peritoneal carcinomatosis, weight of metastatic lesions (mean + SEM) and incidence of ascites fluid in groups of 5–7 mice. Statistically significant differences: panel **B**, p = 0.007 at Student's *t* test; panel **C**, p = 0.027 at Fisher's exact test. (**D**) control mouse, treated with vehicle (PBS) alone, showing multiple i.p. masses (green arrows); (**E**) tumor-free mouse treated with R-LM249; (**F**) distribution of R-LM249 in a mouse bearing multiple i.p. tumors shows that the virus selectively populates and replicates in the HER-2+ masses (green fluorescence: virally-encoded EGFP; yellow-brown florescence: autofluorescence of mouse fur and visceral organs).

Recently, one of our laboratories showed that Rag2^−/−^;Il2rg^−/−^ mice, which lack T, B and NK responses, are superior to nude mice for studies on human tumors [17], [18]. Here, we asked whether the novel model could be employed to evaluate the oncolytic therapy of HER-2+ ovarian carcinoma. SK-OV-3 cells grew i.p. in all Rag2^−/−^;Il2rg^−/−^ mice in a few weeks, and gave also rise to ascitic fluid in most cases; this enabled us to reliably use a fixed end-point of 6 weeks and allowed a quantitative evaluation of peritoneal carcinomatosis (Fig. 3 and Fig. S1). Therapy with R-LM249 produced a strong inhibition of tumor growth, resulting in the absence of ascites in all treated mice and reducing the overall tumor burden by 95% (Figs. 3A–E). EGFP fluorescence, indicative of R-LM249 persistence and expression, hence of virus-susceptible cells, was observed at sacrifice in some residual peritoneal masses of treated mice.

We took advantage of the EGFP sequence engineered in R-LM249 genome to monitor intraperitoneal viral spread and to image the tumors. In this instance, R-LM249 was i.p. administered to mice bearing preformed tumors, i.e. 9 weeks after SK-OV-3 cell injection. The virus populated and replicated in all visible intraperitoneal tumor masses, producing a strong signal (Fig. 3F), practically yielding an *in vivo* imaging of the tumor. EGFP expression was strictly confined to tumor masses, notwithstanding the fact that the amounts of administered virus were about 3 logs higher than those required for wild type virus to kill 100% of mice by the same route [6]. This finding confirms and extends the high specificity of R-LM249 towards HER-2-positive cells [6], and consequently its safety profile. We specify that R-LM249 is retargeted to human HER-2, hence the virus would likely not be able to infect cells expressing endogenous murine HER-2, if they were present. The ability of R-LM249 to infect only cells with a high level of HER-2 cell surface expression [6], argues that the virus is unlikely to infect cells with low level of HER-2, i.e. non cancer cells in which the gene is not amplified.

Cumulatively, the results reported in this section provide the first evidence that R-LM249 exerts anti-tumor activity against peritoneally-spread ovary cancers, and that it is effective following the i.p. systemic route of administration.

### Therapy of subcutaneous HER-2+ breast cancer

**Figure 4:**
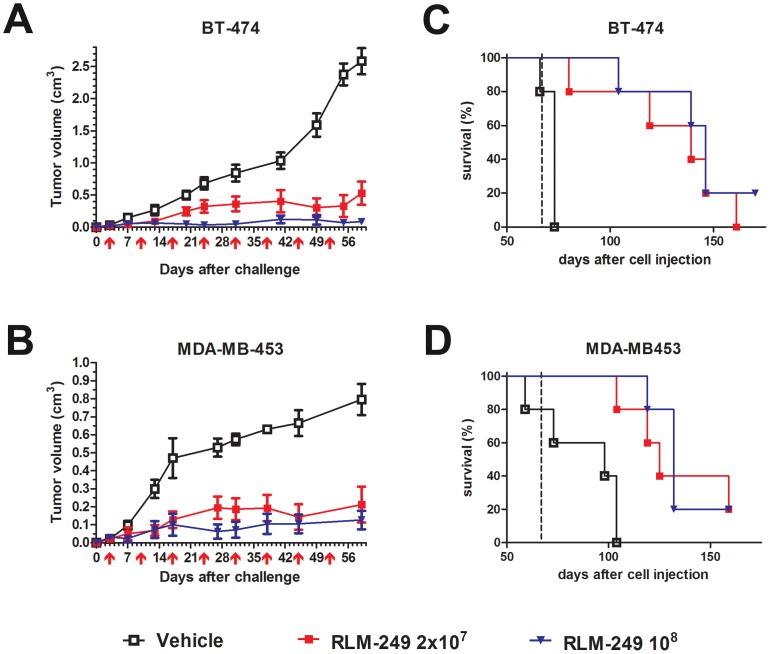
Therapy with R-LM249 of Rag2^−/−^;Il2rg^−/−^ mice bearing s.c. BT-474 or MDA-MB-453 human breast carcinoma cell lines. (**A**) and (**B**), tumor volumes. R-LM249 was administered intratumorally at the indicated amounts (p.f.u.) on the days marked by red arrows below *x*-axis. Mean + SEM of 5 mice per group is shown, until all mice per group are alive. Statistical significance of treatment (at Student's *t* test): panel **A**, p<0.05 at least from day 13 (10^8^ dose) or 20 (2×10^7^ dose); panel **B**: p<0.05 at least from day 13 (both doses). (**C**) and (**D**), Kaplan-Meier analysis. End of therapy is shown by a vertical dashed line. Median survival times (days) for BT-474 groups were: Vehicle = 73, R-LM249 dose 2×10^7^ = 139, dose 10^8^ = 146. Median survival times (days) for MDA-MB-453 groups were: Vehicle = 98, R-LM249 dose 2×10^7^ = 125, dose 10^8^ = 132. Survival curves of treated groups were significantly different from the respective vehicle (p<0.01 at least, Mantel-Haenszel test).

The primary clinical target of therapeutic agents against HER-2 is breast cancer [19]. As a first approach to evaluate the in vivo activity of R-LM249 against HER-2+ breast cancer, we studied the activity of R-LM249 against the growth of subcutaneous tumors induced by human HER-2+ breast cancer cells. The growth of MDA-MB-453 and BT-474 tumors in Rag2^−/−^;Il2rg^−/−^ mice was strongly inhibited by repeated intratumor administrations of R-LM249 (Fig. 4A and B). R-LM249 was effective both at 2×10^7^ and 1×10^8^ PFU/mouse, with significantly higher effects for the higher amount in BT-474 group at several time points, as determined by Student's *t* test. Mice treatment was discontinued at 67 days. Kaplan-Meier analysis showed that the overall survival was significantly extended, with about 20% long-term survivors (Fig. 4C and D). All MDA-MB-453 tumor-bearing mice showed development of brain and lung metastases.

### Therapy of metastases from HER-2+ breast cancer

The visceral organs in the peritoneal cavity are a frequent site of metastatic spread for breast cancer [14]. The metastatic tropism of HER-2+ breast cancer in patients is not readily reproduced in nude mice; recently we reported that Rag2^−/−^;Il2rg^−/−^ mice enabled a multiorgan metastatic spread of MDA-MB-453 and BT-474 cell lines that mirrored the clinical situation, including ovarian, lung and brain metastases in most mice (Fig. S2 and [18]). In this model, metastases are induced through cell injection *via* the intravenous route, thus allowing to mimic an adjuvant therapeutic condition. Here, we took advantage of this novel metastatic model and investigated the therapeutic activity of R-LM249 administered i.p. against metastases of human breast cancer. We focused on MDA-MB-453 cells for two reasons. First, these cells are more metastatic than BT-474 cells. Secondly, their growth is not inhibited by trastuzumab (see Fig. 1B). Hence, they represent a worst case scenario of HER-2+ cancer dissemination.

**Figure 5:**
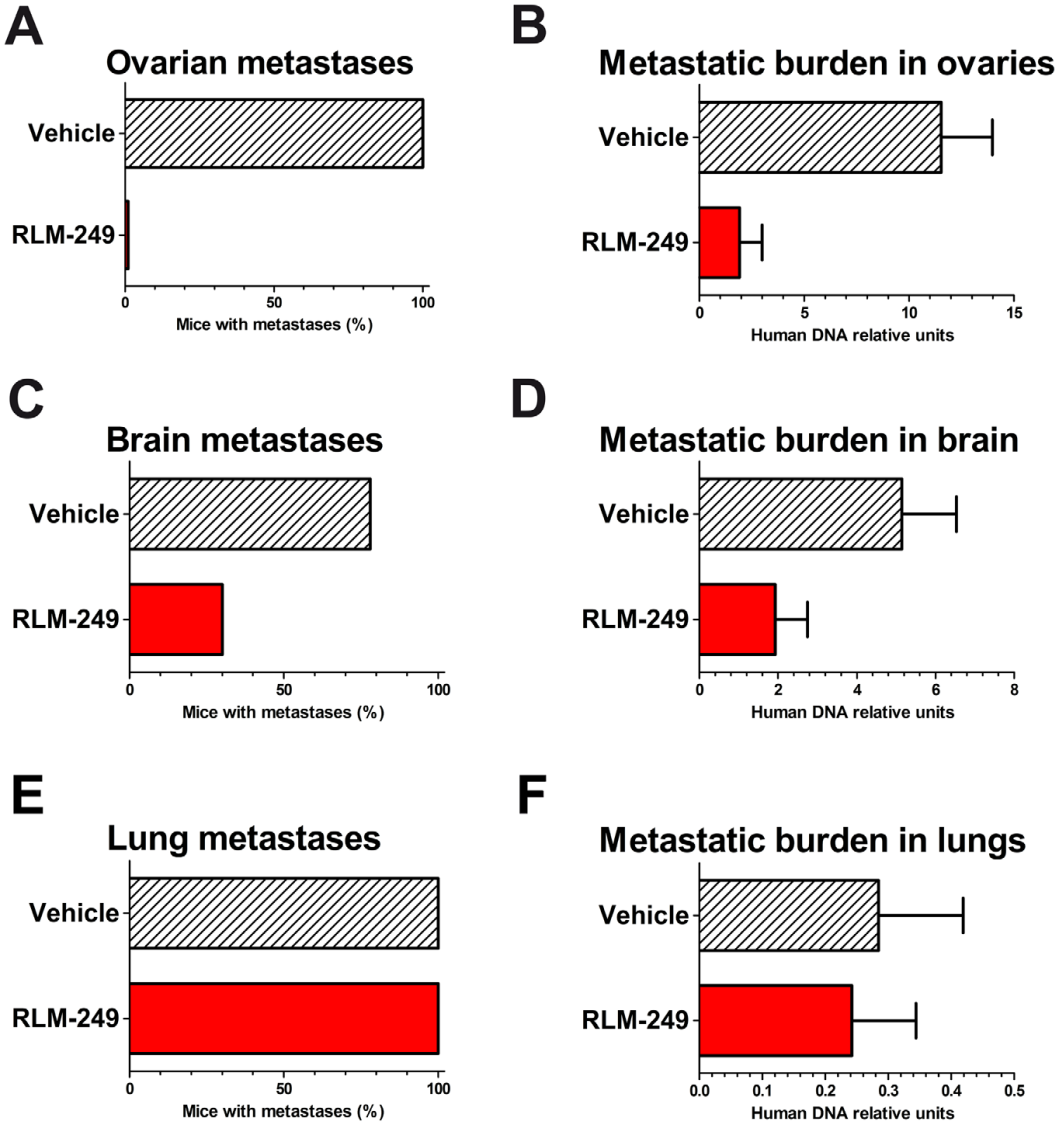
Therapy with R-LM249 of systemic metastases induced by the i.v. injection of human breast carcinoma cell line MDA-MB-453. R-LM249 was administered i.p. at 10^8^ pfu dose (4 weekly injections) to groups of 9–10 Rag2^−/−^;Il2rg^−/−^ mice. (**A, C, E**) incidence of macroscopic metastases in the indicated organs, as determined at necropsy; (**B, D, F**) total metastatic burden (mean + SEM) in organs as quantified by human centromeric DNA qPCR. Statistical analysis of differences: panel **A**, p<0.0001 at Fisher's exact test; panel **B**, p = 0.0004 at both Student's *t* and non-parametric Wilcoxon rank sum tests; panel **C**, p = 0.07 at Fisher's exact test; panel **D**, p = 0.056 at Student's *t* test and p = 0.03 at non-parametric Wilcoxon rank sum test.

All Rag2^−/−^;Il2rg^−/−^ mice inoculated i.v. with MDA-MB-453 breast cancer cells developed large ovarian metastases (Fig. S3), frequently affecting both ovaries. Remarkably, all mice treated i.p. with R-LM249 appeared free from macroscopic ovarian metastases at necropsy (Fig. 5A).

To quantify micrometastases within mouse ovaries we set up a quantitative PCR assay for human centromeric sequences (*see* Materials and Methods). This molecular assay highlighted that the numbers of molecularly-detectable human metastatic cells reached levels significantly lower in the ovaries of virus-treated than in the ovaries of PBS-treated control mice (Fig. 5B). We found a therapeutic activity against brain metastases (Figs. 5C–D) and no effect against lung metastases (Figs. 5E–F).

**Figure 6:**
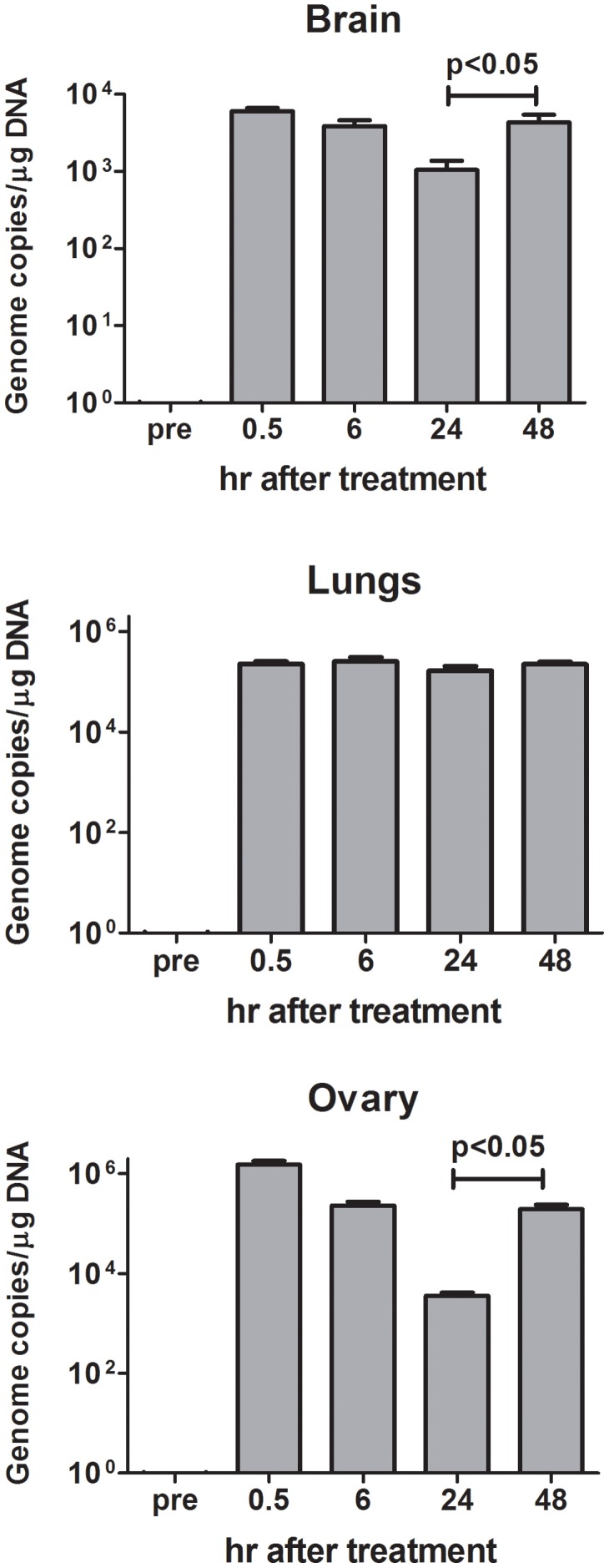
Biodistribution of R-LM249 injected by the intraperitoneal route at 10^8^ pfu in Rag2^−/−^;Il2rg^−/−^ mice bearing MDA-MB-453 metastases. Mean and SEM is shown for 2–3 mice. The number of copies of R-LM249 normalized over the quantity of total DNA extracted from the organ is reported. “pre” corresponds to organs of a mice that did not receive R-LM249.

Since virus replication is dependent on the presence of HER-2+ cells, biodistribution of i.p. administered R-LM249 was studied in mice already bearing MDA-MB-453 metastases. Virus was found in ovaries and lungs and, at lower levels, in brain (Fig. 6), with different kinetics of clearance. Ovaries and brain showed significantly decreased levels up to 24 hr, then virus level increased at 48 hr. This suggests that some viral replication occurred in ovary and brain HER-2^+^ metastatic deposits, in agreement with the above reported therapeutic activity. Kinetics of R-LM249 in lungs was different, with levels unchanged up to 48 hr.

Overall, the i.p.-administered R-LM249 exerted a strong antimetastatic effect against peritoneal metastases of breast cancers. Some evidence of therapeutic effect on extraperitoneal metastases was also found.

## Discussion

Tumor dissemination is the main cause of cancer-related death. A major challenge in oncolytic virotherapy is whether viruses can reach metastatic tumors upon systemic (e.g. intravenous or intraperitoneal) administration. To address this issue, not previously investigated for any retargeted HSVs, we employed mouse models that mirror the intraperitoneal dissemination of the two main HER-2+ cancers, ovarian and breast. This represents an aggressive, advanced form of cancer, associated with dismal prognosis. The HER-2-redirected HSV R-LM249 was administered i.p., to ensure that it readily reached the intraperitoneal metastatic masses. R-LM249 strongly inhibited the peritoneal growth of human HER-2+ trastuzumab-resistant ovarian and breast carcinomas, and its metastatic growth in the brain, but not in the lungs.

### Pertinent to our conclusions is the following

#### (i) HER-2 is a clinically relevant and molecularly attractive target

Different targeting strategies have led to a range of specific drugs against the p185 HER-2 oncoprotein. We envision that the anti-HER-2 virotherapy is not mutually exclusive with the anti-HER-2 specific therapies, rather they can complement each other, or be applied sequentially. The monoclonal antibodies (MAb) and kinase inhibitors (KI) have non-overlapping mechanisms of action, and patients clearly benefit from combinations [20], [21]. An emerging problem is the in vivo selection of cells resistant to the inhibitors. The HER-2 redirected HSV introduces a further antitumor tool, independent of MAb and KI.

The distinct advantage of R-LM249 is its cytotoxic activity independent of tumor cell addiction to HER-2 signaling as well as of host's immunity. Whereas KI are efficacious only if tumor growth and survival are dependent on p185 kinase-mediated signaling, R-LM249 could kill HER-2+ cells independently of p185 kinase activity. In addition to the inhibition of mitogenic signaling, anti-p185 MAbs can kill tumor cells through complement-dependent cytotoxicity or antibody-dependent cell-mediated cytotoxicity (ADCC). Yet, solid tumors, unlike lymphomas and leukemias, are mostly resistant to such immune-mediated lytic mechanisms [22], [23]. R-LM249 can kill HER-2+ cancer cells that are resistant to MAbs, because its cytopathic activity is independent of tumor cell sensitivity to immune-mediated lysis. So far, we have no evidence for *in vitro* selection of cells resistant to R-LM249. As reported in Results, in Rag2^−/−^;Il2rg^−/−^ treated mice some residual small peritoneal masses showed evidence of virus persistence and replication, indicative of virus-susceptible cells. The presence of R-LM249–susceptible cells and of the virus possibly reflects a higher replication rate of the tumor cells relative to that of the virus.

#### (ii) Current data extend the results of a limited number of studies on the efficacy of o-HSV following systemic delivery, with positive indications both in animals and in humans

In mice, i.p. and i.v. administered non-retargeted o-HSVs can indeed hamper the growth of tumor masses [24]–[29], including i.p. SK-OV-3 xenografts [30]. In humans, administration of o-HSV *via* hepatic artery contrasted the growth of metastatic colon cancers in liver [31]–[33]. The o-HSVs employed in the above studies exhibited wt-tropism, hence were capable to infect, and, albeit at reduced level, to replicate and survive at some sites before reaching the tumor. By contrast, retargeted HSVs replicate exclusively after they reach the target tumor and exhaust themselves when they no longer find susceptible cancer cells. Similarly, HER-2-retargeted vesicular stomatitis virus (VSV) administered i.p. could replicate in HER-2+ tumor bearing mice, but not in tumor-free animals [34].

The systemic administration of HER-2-retargeted HSV to humans calls for a high safety profile. *In vivo*, R-LM249 did not kill any mice, even at doses 3 logs higher than those required for wt-virus to kill all mice [6]. A limit of that assay was that R-LM249 is specific to the human HER-2 and likely would not infect cells expressing the murine HER-2. Safety can be inferred by the highly specific tropism of the virus for cells that express HER-2, the requirement for high expression level of the receptor [6], R-LM249 inability to revert to wt-genotype and phenotype consequent to the deletion of a large portion of gD gene, and, finally, the preservation of the thymidine kinase gene that guarantees the efficacy of acyclovir in a worst case scenario.

#### (iii) A remarkable finding was the ability of R-LM249 to reach and populate the tumor masses spread to the peritoneum

This resulted in EGFP expression in every tumor mass, even the small ones, and enabled a visualization of intraperitoneally-spread tumors (Fig. 3F). This property, which rests on the high specificity of the virus, could lead to the development of virus-mediated imaging of tumors, particularly since specific substrates of HSV thymidine kinase were designed, which enable the detection of HSV-infected cells by the non invasive, repeatable positron emission tomography (PET) [35].

#### (iv) The effect of i.p.-administered R-LM249 against brain metastases most likely reflects the R-LM249 ability to reach the brain tumors, documented here by detection of HSV genome copies in the brain (Fig. 6)

Alternatively, it may be consequent to the reduction in peritoneal tumor growth, should the brain metastases originate from tumor cells leaving the peritoneal cavity. The differential effect of R-LM249 against metastasis in different organs could be consequent to microenvironmental interactions. R-LM249 reached and persisted in lungs, but was not effective against lung metastases. The high macrophage content of lungs could play a role [36].

In previous studies we identified two privileged positions in gD which can accept single chain antibodies and enable to engineer fully retargeted o-HSVs [4], [5]. A strong advantage of the technology is that the HER-2-HSVs can be easily re-engineered to target different oncoproteins, through the replacement of the trastuzumab-encoding cassette with sequences encoding any other single-chain antibody, or with sequences encoding natural ligands to the selected cancer-specific receptors [2]. Thus, the efficacy of systemically administered retargeted o-HSV reported here will no doubt impact on the choice of additional targets and on future developments of retargeted o-HSV.

## Materials and Methods

### Ethics statement

All animal experiments were performed according to Italian law 116/92 and European directive 2010/63/UE. Experimental protocols were reviewed and approved by the Institutional Animal Care and Use Committee (“Comitato Etico Scientifico per la Sperimentazione Animale”) of the University of Bologna, and forwarded to the Italian Ministry of Health with letters 19413-X/10 (Responsible Researcher Prof. C. De Giovanni) and 20600-X/10 (Responsible Researcher Prof. P.-L. Lollini).

### Cells

Human ovarian cancer cell line SK-OV-3 and human breast cancer cell lines MDA-MB-453 and BT-474 (all from American Type Culture Collection, ATCC) were kindly given by Dr. Serenella M. Pupa (Istituto Nazionale dei Tumori, Milan, Italy). Cell lines were authenticated by DNA fingerprinting on the 11^th^ November 2010 (performed by DSMZ, Braunschweig, Germany). In selected *in vitro* experiments the MDA-MB-231 cell line (ATCC) was also used. Cells were cultured in RPMI+10% FBS at 37°C in a humidified 5% CO_2_ atmosphere.

### Mice

Rag2^−/−^;Il2rg^−/−^ breeders were kindly given by Drs. T. Nomura and M. Ito of the Central Institute for Experimental Animals (Kawasaki, Japan). Mice were then bred in our animal facilities under sterile conditions and used for the experiments described here at 10–14 weeks of age. Athymic Crl:CD-1-*Foxn1* ^nu/nu^ mice (referred to as nude mice) 6-week-old were purchased from Charles River Italy and kept under sterile conditions.

### RLM-249 oncolytic virus

R-LM249 virus was grown in SK-OV-3 and titrated in SK-OV-3 cells by plaque assay. All experiments were carried out with partially purified extracellular viruses harvested by high speed centrifugation.

### Cytotoxicity assay

To evaluate the cytotoxic effect of R-LM249, cells were seeded in 96 well plates at 8×10^3^ cell/well and infected with R-LM249 (10 pfu/cell) or mock-infected. AlamarBlue (10 µl/well, Life Technologies) was added to the culture media at 48, 96 and 144 h after infection and incubated for 4 h at 37°C. Plates were read at 570 and 600 nm with a Synergy HTTR-I fluorometer (BioTek). For each time point, cell viability was expressed as the percentage alamarBlue reduction in infected *versus* uninfected cells, excluding for each set of samples the contribution of medium alone.

### Sensitivity to trastuzumab

Cells were seeded in 96 well plates at 5–10×10^3^ cell/well and let to adhere overnight. Trastuzumab (Genentech Inc.) was then added to each well at final concentrations ranging 0.001–100 µg/ml and cultures were incubated for further 72–120 hr. Cell growth and viability were determined after 1 hr incubation with 1/10 volume of Cell Proliferation Reagent WST-1 (Roche Applied Science), reading absorbance on an ELISA plate reader with a test wavelength at 450 nm and a reference wavelength at 630 nm. Results were expressed as percentage of untreated controls.

### Peritoneal carcinomatosis of ovarian carcinoma

For the induction of ovarian cancer peritoneal carcinomatosis, nude mice or Rag2^−/−^;Il2rg^−/−^ mice received 2×10^6^ ovarian carcinoma SK-OV-3 cells intraperitoneally (i.p.). R-LM249 was administered weekly (for five weeks) at 2×10^7^–10^8^ plaque-forming units, starting 3 days after the injection of tumor cells. Nude mice were sacrificed depending on tumor growth. For Rag2^−/−^;Il2rg^−/−^ mice, preliminary experiments showed that tumor masses could be clearly observed as early as 6 weeks after tumor cell injection. Accurate autopsy was performed with the aid of low-magnification device and intraperitoneal tumor masses were collected and weighted to quantify therapeutic efficacy.

### Breast carcinoma models

The efficacy of R-LM249 against human HER-2^+^ breast carcinoma cells was tested in two experimental systems, i.e. against the growth of local tumors and against metastases.

For the induction of tumors, cell line MDA-MB-453 was injected subcutaneously (2×10^6^ cells) in a flank of Rag2^−/−^;Il2rg^−/−^ mice. R-LM249 was administered weekly (for ten weeks) at 2×10^7^–10^8^ plaque-forming units, starting 3 days after the injection of tumor cells, as detailed in figure legend. At this time most mice showed very small but palpable masses, mice were randomized in the different groups based on tumoral mass size. Quantification of s.c. tumor masses was assessed weekly (just before R-LM249 administration) by measuring with a caliper, tumor volume was calculated as π·[√(*a*·*b*)]^3^/6, where *a* = maximal tumor diameter, and *b* = tumor diameter perpendicular to *a* [6].

For the induction of metastases, cell line MDA-MB-453 was injected intravenously (i.v.) (2×10^6^ cells). R-LM249 was administered weekly (for four weeks) at 2×10^7^–10^8^ plaque-forming units, starting 3 days after the injection of tumor cells. Mice were sacrificed 10 weeks after tumor cell injection. Incidence of macroscopic metastases was determined at necropsy. Metastatic burden was estimated by human centromeric DNA qPCR quantification on genomic DNA of dissected organs (ovaries, brain and lungs). Human centromeric DNA qPCR quantification of metastatic burden in dissected organs (ovaries, brain and bone marrow) was performed by Real-Time PCR performed on genomic DNA, extracted with 10 mM Tris-HCl buffer pH 8.3 containing 50 mM KCl, 2.5 mM MgCl_2_, 0.01% gelatin, 0.45% Igepal, 0.45% Tween 20 and 120 µg/ml proteinase K (all reagents from Sigma, Milan, Italy) by an overnight incubation at 56°C followed by 30 min incubation at 95°C to inactivate the proteinase K. A sequence of the α-satellite region of the human chromosome was amplified. Primer and probe sequences derived from Becker et al. [37] with the sole alteration that the probe carried the non-fluorescent quencher dye TAMRA at the 3′-end. A 100 ng DNA aliquot per sample was amplified using 250 nM primers and 100 nM probe in a final volume of 25 µl of TaqMan Universal PCR Master Mix (Applied Biosystems, Milan, Italy). After an initial denaturation step at 95°C for 10 min, 45 cycles of amplification (95°C for 30 sec plus 60°C for 1 min) were performed in a 5700 Sequence Detection System (Applied Biosystems). DNA extracted from mouse tissues showed no amplification up to 45 cycles. To quantify human cells, standard curves were constructed by adding serial amounts of MDA-MB-453 human cells (from 100% to 0.001%) to fixed amounts of mouse cells or mouse whole brain. C_t_ (threshold cycle) values obtained from the experimental samples were interpolated in the standard curve run in each PCR to obtain the relative amount of human to murine cells in the sample (referred to as human DNA relative units). Three to five independent determinations were performed for each specimen.

### Biodistribution of R-LM249

R-LM249 was injected i.p. at 10^8^ pfu in 0.4 ml in Rag2^−/−^;Il2rg^−/−^ mice bearing metastases from MDA-MB-453 (7 weeks after the i.v. injection of cells as reported above). Mice were sacrificed at various times (0.5, 6, 24 and 48 hr after virus injection). Ovaries, lungs and brain were collected at sacrifice and immediately frozen. DNA was then extracted with NucleoSpin Tissue (Macherey-Nagel) accordingly to the manufacturer's instruction and subjected to qPCR, with a standard curve of known quantities R-LM249 (from 25000 to 8 viral genome copies) performed in parallel. Primers used were: gG_504_f CTTGGTTCCGACGCCTCAACATAC and gG_603_r TAAGGTGTGGATGACGGTGCTGAC.

### Statistical analysis

Tumor-free survival curves (Kaplan–Meier) were compared by the Mantel-Haenszel test. Tumor volumes and tumor or metastatic burdens were compared by the Student's *t* test and the non-parametric Wilcoxon rank sum test. Frequencies of mice with metastases were compared by Fisher's exact test.

## Supporting Information

Figure S1 Dissemination of human SK-OV-3 ovarian carcinoma in the peritoneal cavity of Rag2^−/−^;Il2rg^−/−^ mice. Each panel shows multiple tumor nodules in one mouse 6 weeks after an i.p. challenge with 2×10^6^ cells. (**A**) Multiple nodules adhering to the omentum between liver and stomach; (**B**) Large nodules underneath the liver; (**C**) three nodules in the lower peritoneal cavity (detail of mouse of Figure 3d); (**D**) a large mass behind uterine horns.
(TIF)

Figure S2 Intracerebral MDA-MB-453 metastases in Rag2^−/−^;Il2rg^−/−^ mice evidenced by immunohistochemical staining for HER-2 (in brown). Mice received the i.v. injection of 2×10^6^ MDA-MB-453 cells. Numerous metastases preferentially localised in the cerebral cortex were observed. Most metastases initially appear as intravascular metastases (**A**) invading the brain parenchyma by direct extension (**C**) or along blood vessels (**B**, **D**).
(TIF)

Figure S3 Ovarian metastasis in a Rag2^−/−^;Il2rg^−/−^ mouse 10 weeks after the i.v. injection of 2×10^6^ MDA-MB-453 breast cancer cells.
(TIFF)
