# Supplementary figures and images for: Preclinical Therapy of Disseminated HER-2+ Ovarian and Breast Carcinomas with a HER-2-Retargeted Oncolytic Herpesvirus
Source: PLoS Pathog. 2013 Jan 31;9(1):e1003155. doi: 10.1371/journal.ppat.1003155 (PMC3561254; doi:10.1371/journal.ppat.1003155)

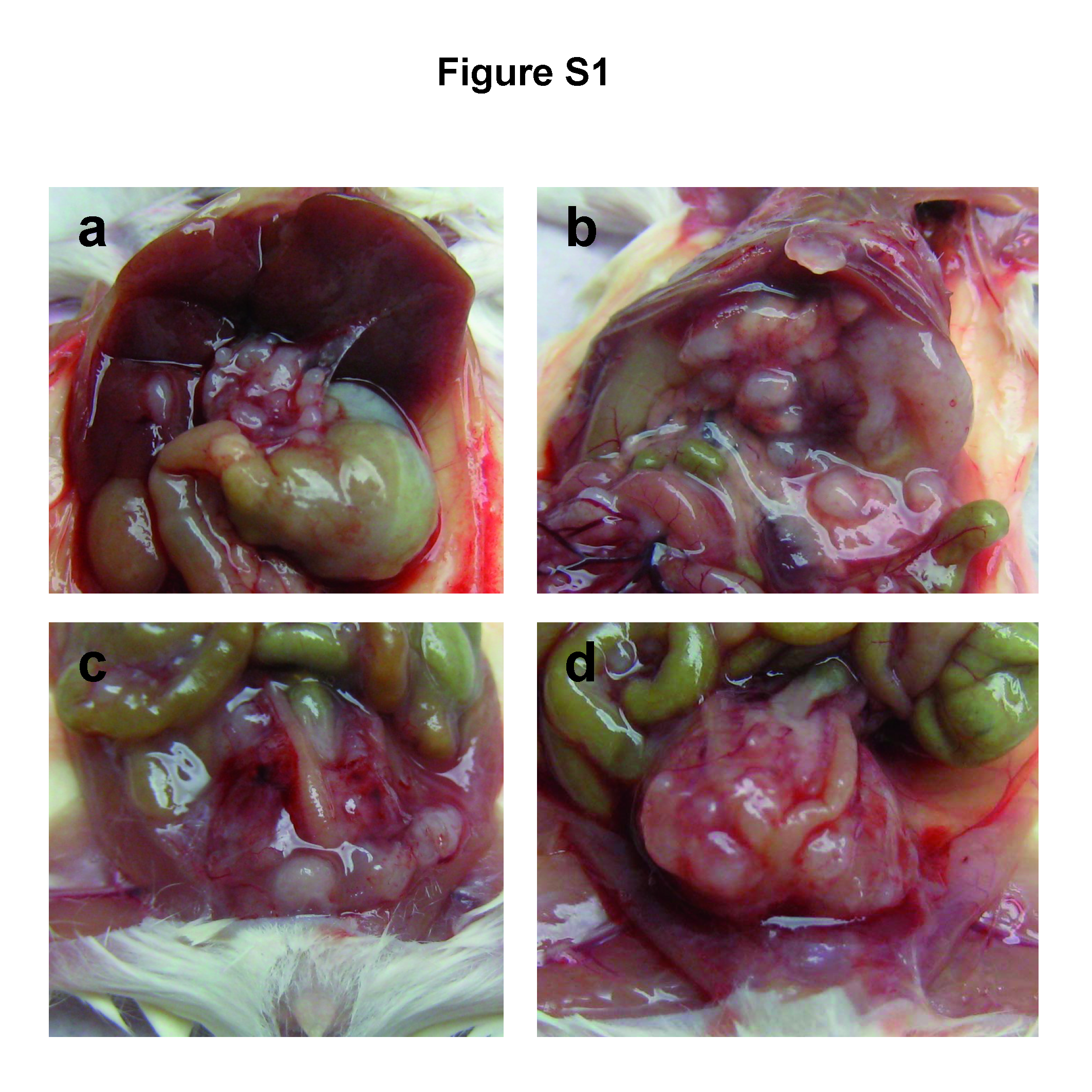

Supplement: Figure S1 — Dissemination of human SK-OV-3 ovarian carcinoma in the peritoneal cavity of Rag2−/−;Il2rg−/− mice. Each panel shows multiple tumor nodules in one mouse 6 weeks after an i.p. challenge with 2×10^6^ cells. (A) Multiple nodules adhering to the omentum between liver and stomach; (B) Large nodules underneath the liver; (C) three nodules in the lower peritoneal cavity (detail of mouse of Figure 3d); (D) a large mass behind uterine horns. (TIF) [file ppat.1003155.s001.tif]

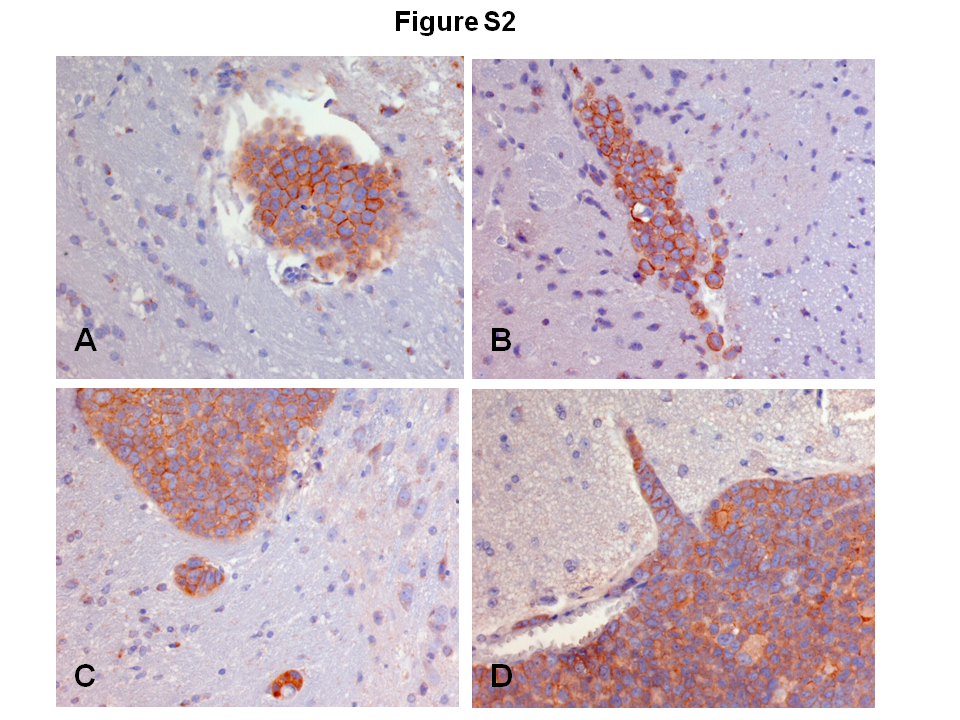

Supplement: Figure S2 — Intracerebral MDA-MB-453 metastases in Rag2−/−;Il2rg−/− mice evidenced by immunohistochemical staining for HER-2 (in brown). Mice received the i.v. injection of 2×10^6^ MDA-MB-453 cells. Numerous metastases preferentially localised in the cerebral cortex were observed. Most metastases initially appear as intravascular metastases (A) invading the brain parenchyma by direct extension (C) or along blood vessels (B, D). (TIF) [file ppat.1003155.s002.tif]

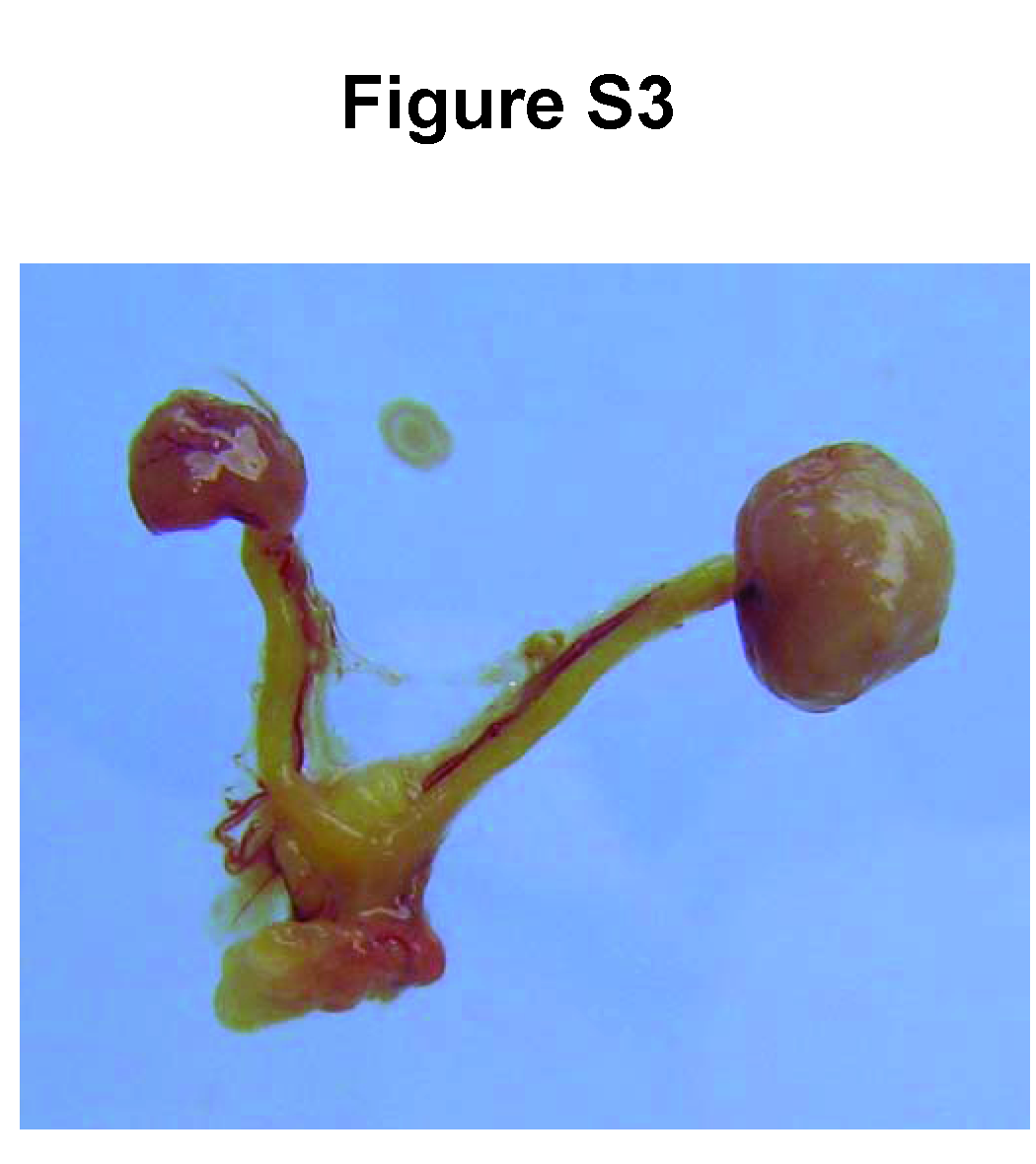

Supplement: Figure S3 — Ovarian metastasis in a Rag2−/−;Il2rg−/− mouse 10 weeks after the i.v. injection of 2×10^6^ MDA-MB-453 breast cancer cells. (TIFF) [file ppat.1003155.s003.tiff]
